# Detecting Phase Transitions from Data Using Generative Learning

**DOI:** 10.3390/e28040406

**Published:** 2026-04-03

**Authors:** Xiyu Zhou, Yan Mi, Pan Zhang

**Affiliations:** 1 School of Fundamental Physics and Mathematical Sciences, Hangzhou Institute for Advanced Study, UCAS, Hangzhou 310024, China; zhouxiyu23@mails.ucas.ac.cn; 2 University of Chinese Academy of Sciences, Beijing 100049, China; miyan@itp.ac.cn; 3 Institute of Theoretical Physics, Chinese Academy of Sciences, Beijing 100190, China

**Keywords:** phase transition, machine-learning, variational autoregressive networks, generative learning

## Abstract

Identifying phase transitions in complex many-body systems traditionally necessitates the definition of specific order parameters, a task often requiring prior knowledge of the statistical model and the symmetry-breaking mechanism. In this work, we propose a framework for detecting phase transitions directly from raw (experimental) data without requiring knowledge of the underlying model Hamiltonian, parameters, or pre-defined labels. Inspired by generative modeling in machine learning, our method utilizes autoregressive networks to estimate the normalized probability distribution of the system from raw configuration data. We then quantify the intrinsic sensitivity of this learned distribution to control parameters (such as temperature) to construct a robust indicator of phase transitions. This indicator is based on the expectation of the change in absolute logarithmic probability, derived entirely from the raw data. Our approach is purely data-driven: it takes raw data across varying control parameters as input and outputs the most likely estimate of the phase transition point. To validate our approach, we conduct extensive numerical experiments on the 2D Ising model on both triangular and square lattices, and on the Sherrington–Kirkpatrick (SK) model utilizing raw data generated via Markov Chain Monte Carlo and Tensor Network methods. The results demonstrate that our generative approach accurately identifies phase transitions using only raw data. Our framework provides a general tool for exploring critical phenomena in model systems, with the potential to be extended to realistic experimental data where theoretical descriptions remain incomplete.

## 1. Introduction

Identifying the phases of matter and their transitions stands as an important research topic in statistical and condensed-matter physics. Critical phenomena, marking the transition between ordered and disordered phases in statistical physics, are central to our understanding of complex systems [1]. A classic example of a phase transition is the paramagnetic–ferromagnetic transition in the Ising model, which occurs when a control parameter (temperature) decreases across a critical value. During this transition, spontaneous symmetry breaking takes place, and the order parameter—magnetization—shifts from zero to a finite value. Traditional approaches to studying these transitions rely heavily on the Ginzburg–Landau–Wilson paradigm [2], which necessitates the identification of a specific order parameter (e.g., magnetization) to characterize symmetry breaking. However, for systems exhibiting topological order or competing interactions, such as spin glasses, determining the appropriate order parameter is non-trivial and often requires profound physical intuition. Moreover, in the more realistic scenario where only experimental data is available, i.e., without prior knowledge of the type or location of a potential phase transition, order parameters cannot be defined a priori.

Recently, machine learning (ML) has emerged as a powerful toolkit for phase transition detection, offering a data-driven alternative to traditional methods. Supervised learning approaches treat phase detection as a classification task, mapping configurations to phase labels [3,4,5]. While effective, supervised learning requires labeled training data, which inherently introduces human bias and assumes prior knowledge of the phase diagram. To mitigate this, label-free methods like Learning by Confusion [6,7] have been developed. Learning by Confusion identifies transitions by monitoring the performance dip of a classifier. Beyond classification tasks, Prediction-Based Methods [8] treat the control parameter (e.g., temperature) as a regression target, and the vector field divergence of outputs is used to search for phase boundaries.

Despite their success, these methods share a common limitation: they are primarily discriminative. They focus on distinguishing between phases or predicting parameters, effectively treating the underlying probability distribution of the system as a “black box”. Consequently, the rich thermodynamic information encoded in the probability density of the configurations is often overlooked. Furthermore, these methods often implicitly rely on the assumption that distinct phases can be separated by a hyperplane or a regression curve, which may not capture the full complexity of the transition.

In recent years, the primary unsupervised learning methods for phase transition detection include principal component analysis (PCA) and autoencoder [9,10]. As one of the most widely used approaches with computational simplicity, principal component analysis solves for the set of orthogonal eigenvectors of the positive semi-definite covariance matrix via linear transformations, such as singular value decomposition (SVD). Phase transitions are then detected by analyzing the projections of input data onto the principal components [11,12,13,14]. However, principal component analysis is constrained by its reliance on linear transformations of data, rendering it challenging to identify nonlinear critical fluctuations. Diffusion maps (DM) is an unsupervised manifold learning algorithm that constructs diffusion affinities via nonlinear operations and maps high-dimensional data onto a low-dimensional manifold, where different phases of the system can be clearly distinguished [15,16]. Another interesting unsupervised approach centers on Score-Based Generative Models (SBMs). Using only limited data from stable phases, it can generate samples of complex many-body systems at arbitrary temperatures from a simple prior distribution, thereby enabling the inference of phase transition behaviors and critical exponents [17]. A nonlinear transformation approach is the autoencoder, which consists of an encoder and a decoder. This framework compresses data into a lower-dimensional representation and reconstructs the original data, treating the disorder metrics as reconstruction targets and detecting phase transitions via reconstruction error of decoded inputs [18,19]. As further optimized in [20], the standard deviation of the loss function is employed to identify distinct phases. However, such methods—characterized by detecting anomalies to reveal underlying patterns—may capture spurious correlations and lack strong physical interpretability.

In this work, we propose a shift from discriminative to generative learning for identifying phase transitions. Our core insight is that a phase transition represents a drastic reorganization of the system’s configuration space. Therefore, by directly modeling the probability distribution of the system, we can detect transitions by monitoring the intrinsic changes in this distribution. We employ autoregressive networks as our generative framework [21,22]. Unlike other generative models, autoregressive networks offer a distinct advantage crucial for physics: tractability. They can explicitly compute the normalized probability, p(s), for any given configuration s.

Crucially, our framework is purely data-driven and model-agnostic. It requires neither prior knowledge of the system’s hamiltonian nor pre-defined labels or order parameters. As long as a dataset of raw configurations (snapshots) is provided—whether from numerical simulations or physical experiments—our method can operate effectively. This universality makes it particularly valuable for studying systems where the theoretical description is incomplete or where the energy function is unknown.

Building on the tractable likelihood provided by autoregressive networks, we introduce a robust, unsupervised indicator to detect phase transitions. Instead of using standard divergence measures that may suffer from sign cancellations during summation, we define a specific indicator that accumulates the absolute magnitude of the logarithmic probability changes between neighboring parameter points. This formulation ensures that all local fluctuations in the configuration space contribute positively to the detection signal, allowing us to capture the total sensitivity of the distribution to parameter tuning. We demonstrate the effectiveness of our approach on the 2D Ising model and Sherrington–Kirkpatrick (SK) model [23] using data from both Monte Carlo simulations and Tensor Network sampling. Our method accurately identifies critical points solely from raw configurations, providing a powerful tool for exploring phase transitions in a strictly unsupervised manner.

## 2. Methodology

### 2.1. Divergence-Based Indicator

In this study, we employ a divergence-based indicator to detect phase transitions solely from sampled data $T=\{s_{1},\;s_{2},\;...,\;s_{\left|T\right|}\}$. Let the system’s configurations be sampled under a scalar control parameter *t* (e.g., temperature in physical systems or a generic tuning variable in other contexts). Our goal is to identify critical points by monitoring the distributional changes driven by *t*.

Leveraging the properties of the Kullback–Leibler (KL) divergence in quantifying distributional proximity [24], we begin with the symmetric difference of the KL divergence, denoted as ΔKL. Expanding its definition yields a weighted summation over the full configuration space:

(1) $$\Delta KL=\sum_{s}p_{t}\left(s\right)\ln\frac{p_{t+\delta t}\left(s\right)}{p_{t-\delta t}\left(s\right)}.$$

While ΔKL theoretically characterizes the net drift of the distribution, the logarithmic term represents a signed rate of change. Consequently, contributions from different configurations may cancel each other out during the summation (i.e., sign cancellation), potentially masking the signal of critical fluctuations where the distribution undergoes rigorous reorganization but little net shift.

To robustly capture the total magnitude of the distributional sensitivity, we define our specific indicator I(t) by incorporating an absolute value into the accumulation. Furthermore, since the explicit summation in (1) is computationally intractable for high-dimensional systems, we approximate the indicator as an expectation over the sampled data $p_{t}\left(s\right)$:

(2) $$I\left(t\right)=E_{s∼T}\left[\left|\ln\frac{p_{t+\delta t}\left(s\right)}{p_{t-\delta t}\left(s\right)}\right|\right].$$

This formulation ensures that all local probability shifts contribute positively to the detection signal. Additionally, by operating on logarithmic probability ratios rather than raw probabilities, this approach inherently mitigates numerical underflow issues. Several other information-theoretic measures are detailed in Appendix A.

To evaluate the indicator, explicit knowledge of the probability configuration p(s) is required. Since the true distribution underlying the sampled data is unknown—and indeed, its analytical form is not required—we approach this as a density estimation problem. Therefore, a parameterized variational distribution $q_{\theta }\left(s\right)$ is introduced to approximate the true distribution, where θ denotes the learnable parameters.

The optimization objective is to maximize the log-likelihood of the model over the observed dataset T. Mathematically, this is equivalent to minimizing the KL divergence between the data distribution and the model distribution:

(3) $$D_{\mathrm{KL}}\left(p\left(s\right)|\right|q_{\theta }\left(s\right))=\sum_{s}p\left(s\right)\ln p\left(s\right)-\sum_{s}p\left(s\right)\ln q_{\theta }\left(s\right),$$

Since the first item is independent of the parameters, the optimal parameters $\theta^{*}$ are obtained by

(4) $$\theta^{*}=\underset{\theta }{\mathrm{argmin}}L\left(\theta \right)=\underset{\theta }{\mathrm{argmin}}E_{s∼T}\left[-\ln q_{\theta }\left(s\right)\right],$$

where the loss function L(θ) is often called cross-entropy or negative log-likelihood. Upon convergence, the optimized model $q_{\theta^{*}}\left(s\right)$ serves as an accurate surrogate for p(s), allowing for the calculation of the indicator I(t).

We use density estimation to compute the normalized configuration probabilities of an unknown system, and locate potential critical points for phase transitions by analyzing the sensitivity of the probability distribution to the control parameter, without relying on prior system knowledge or an explicit order parameter.

### 2.2. Autoregressive Networks

We implement the variational distribution $q_{\theta }\left(s\right)$ using autoregressive networks [22]. Distinguished by their tractability, autoregressive networks enable both efficient sampling and, crucially, the computation of the exact normalized probability for any given configuration. This is achieved by decomposing the high-dimensional joint distribution into a product of conditional probabilities via the chain rule [25,26]:

(5) $$q_{\theta }\left(s\right)=\prod_{i=1}^{N}q_{\theta }\left(s_{i}|s_{1},\ldots ,s_{i-1}\right).$$

The architecture of the autoregressive network is selected based on the dimensionality and spatial structure of the target data. For simple one-dimensional sequential data, fully connected layers with autoregressive masks are sufficient to capture dependencies. For high-dimensional data with spatial topology, such as two-dimensional lattice configurations or images, the PixelCNN architecture is typically employed to preserve local spatial correlations [27,28].

PixelCNN leverages masked convolutions to model the conditional probability of a site based on its preceding context, effectively strictly enforcing the autoregressive property on 2D grids. Despite its effectiveness, standard PixelCNN architectures suffer from the blind spot problem due to the geometric constraints of the receptive field [29]. Specifically, standard masked convolutions may fail to incorporate information from certain visible regions (e.g., the upper-right context in a raster scan), leading to information-deficient predictions.

**Figure 1:**
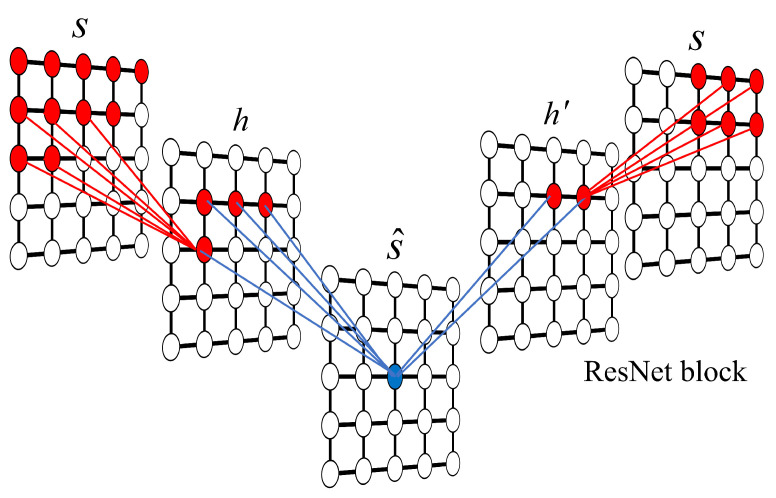
Schematic of the two-dimensional autoregressive network with ResNet blocks. The input configuration s is processed to produce the output $\hat{s}$. Here, h denotes standard hidden layers, while $h^{\prime }$ represents the hidden layers within the ResNet block. The colored regions illustrate the effective receptive field for a target site in $\hat{h}$. Connections are simplified for clarity.

To mitigate this issue and capture long-range correlations effectively, we enhance the network by integrating Gated Residual Blocks (ResNet blocks) with masks, as illustrated in Figure 1. The inclusion of residual connections allows for deeper network structures, which significantly expands the receptive field, thereby eliminating blind spots and ensuring that the probability prediction for each site utilizes the complete available historical context.

## 3. Numerical Experiments

To demonstrate the efficacy of our method in detecting phase transitions in finite-size statistical mechanics systems, we perform numerical experiments on the two-dimensional (2D) ferromagnetic Ising model applied to both triangular and square lattices.

The 2D Ising model is defined on a lattice of N=L×L sites. The state of the system is described by a configuration vector $s=(s_{1},s_{2},\ldots ,s_{N})$, where each spin variable takes a discrete value $s_{i}\in \left\{+1,-1\right\}$. The system is governed by the Boltzmann distribution [30,31,32]:

(6) $$p\left(s\right)=\frac{1}{Z}e^{-\beta E(s)},$$

where $Z=\sum_{s}e^{-\beta E(s)}$ is the partition function, and β=1/T is the inverse temperature (setting the Boltzmann constant $k_{B}=1$). The Hamiltonian (energy function) for a given configuration s is defined as

(7) $$E\left(s\right)=-J\sum_{\langle i,j\rangle }s_{i}s_{j},$$

where the summation $\sum_{\langle i,j\rangle }$ runs over all nearest-neighbor pairs, and J>0 represents the ferromagnetic interaction strength.

This model undergoes a well-known second-order phase transition characterized by spontaneous symmetry breaking (SSB). Below the critical temperature $T_{c}$, the interaction strength dominates thermal fluctuations, inducing a spontaneous alignment of spins and resulting in a nonzero spontaneous magnetization M(T). Conversely, above $T_{c}$, thermal fluctuations destroy this long-range order, driving the system into a disordered paramagnetic phase where the magnetization vanishes.

In our experiments, the autoregressive network is trained to approximate the distribution of sampled data. We employ the Adam optimizer for minimizing the loss function, and all computational results presented below are obtained using the NVIDIA A100 GPU (NVIDIA Corporation, Santa Clara, CA, USA) devices.

### 3.1. 8 × 8 Triangular-Lattice Ferromagnetic Ising Model

We first validate our indicator on the ferromagnetic Ising model defined on an 8×8 triangular lattice with periodic boundary conditions. For this specific geometry, the exact spontaneous magnetization M(T) is analytically known [33]:

(8) $$M{\left(T\right)}^{8}=1-\frac{16x^{6}}{\left(1+3x^{2}\right){\left(1-x^{2}\right)}^{3}},$$

where $x=e^{-2J/T}$. This analytical solution indicates that the magnetization vanishes continuously as the temperature approaches the critical point $T_{c}=4J/\ln3$.

To implement the phase transition detection, we generated unbiased configuration samples using the standard Metropolis–Hastings MCMC algorithm [34,35]. The dataset covers a temperature range encompassing the critical point, consisting of 9 distinct temperature points with a spacing of δT=0.4. At each temperature, $2\times 10^{4}$ independent samples were collected to ensure statistical convergence.

Given the moderate system size ($N=L^{2}=64$), the MCMC sampling effectively explores the configuration space. The network architecture consists of 4 convolutional layers with 64 channels and a kernel radius of k=2. This setup satisfies the receptive field condition d×k+1≥L [22], ensuring that the autoregressive property covers the correlations across the entire lattice.

**Figure 2:**
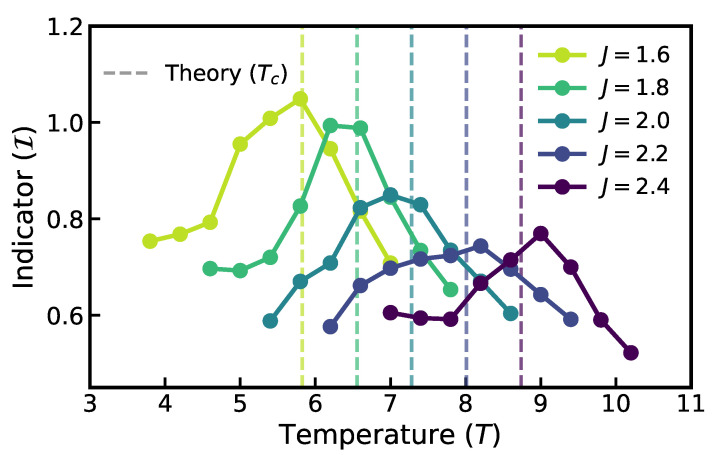
Detection results for the 8×8 triangular-lattice Ising model under varying interaction strengths. The colored curves represent the indicator I(T) computed for different coupling constants J∈{1.6,1.8,2.0,2.2,2.4}. The vertical dotted lines mark the corresponding theoretical critical temperatures $T_{c}\left(J\right)=4J/\ln3$. The systematic alignment of the indicator peaks with the theoretical shifts validates the method’s capability to capture phase boundaries across different energy scales.

The trained autoregressive network provides the estimated probability densities required to calculate the indicator I(T) as defined in (2). The results are presented in Figure 2. To strictly test the robustness of our indicator, we varied the ferromagnetic interaction strength *J* from 1.6 to 2.4 in steps of 0.2. As the interaction strength increases, the energy barrier against thermal fluctuations rises, theoretically shifting the critical temperature $T_{c}$ to higher values.

Our divergence-based indicator I(T) accurately captures this dependency. As shown in Figure 2, the peak of the indicator shifts systematically to the right as *J* increases. For instance, at J=1.6, the indicator peaks at T=5.8, closely matching the theoretical critical point $T_{c}\approx 5.83$. Similarly, at the strongest coupling J=2.4, the peak is located at T=8.6, consistent with the theoretical prediction of $T_{c}\approx 8.74$ (given the discrete sampling interval of δT=0.4). Across all five independent MCMC chains representing different energy scales, the indicator’s maximum consistently aligns with the theoretical phase boundary, demonstrating the method’s universality and its ability to detect phase transitions without prior knowledge of the coupling constants.

### 3.2. 16 × 16 Square-Lattice Ferromagnetic Ising Model

We further extend our investigation to a larger system: the 16×16 square-lattice ferromagnetic Ising model (J=1) with open boundary conditions. The theoretical spontaneous magnetization M(T) for the infinite square lattice is given by Yang [36]:

(9) $$M{\left(T\right)}^{8}=1-\frac{16x^{4}}{{\left(1-x^{2}\right)}^{4}},$$

where $x=e^{-2J/T}$. The critical temperature for the phase transition in the thermodynamic limit is determined by the Onsager solution [32]:

(10) $$T_{c}=\frac{2}{\ln(1+\sqrt{2})}\approx 2.269,$$

corresponding to a critical inverse temperature $\beta_{c}=1/T_{c}\approx 0.4407$.

For large systems, a prominent limitation of the MCMC algorithm is critical slowing down—particularly near the critical points of phase transitions. Owing to the low efficiency of local update schemes, the number of required thermalization steps exhibits power-law, or even exponential, growth. To ensure high-fidelity data for this density estimation task, we utilize Projected Entangled Pair States (PEPSs) [37,38], a Tensor Network method capable of generating uncorrelated samples with high precision as detailed in Appendix B. The dataset spans an inverse temperature range of β∈[0.2,0.7]. To capture the rapid fluctuations near the phase transition, we performed dense sampling with a step size of δβ=0.02 around $\beta_{c}$, while a coarser step of δβ=0.05 was used elsewhere. Each parameter point contains 20,000 samples, split evenly into training and testing sets.

**Figure 3:**
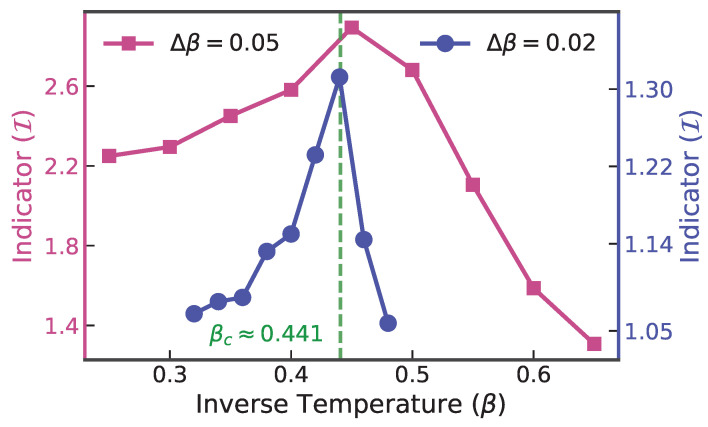
Detection results for the 16×16 square-lattice Ising model. The indicator is calculated over a range of inverse temperatures β∈[0.2,0.7]. The dataset includes a coarse scan (δβ=0.05) and a refined scan (δβ=0.02) near the critical point. All configuration samples were generated using Projected Entangled Pair States (PEPSs) [37]. The dashed line indicates the theoretical critical point $\beta_{c}\approx 0.4407$.

For this configuration, the autoregressive network architecture was adjusted to 3 convolutional layers with 64 channels and a kernel radius of k=5 to accommodate the spatial correlations of the square lattice. The indicator I(β) was then computed for all β. The detection results are illustrated in Figure 3. To simulate a realistic experimental scenario where the critical point is initially unknown, we adopted a hierarchical scanning strategy. First, we performed a coarse scan over a broad temperature range with a step size of δβ=0.05 (red squares). The indicator I(β) reveals a broad region of high sensitivity, reaching a maximum at β=0.45.

Guided by this coarse signal, we then performed a refined scan with a higher resolution of δβ=0.02 (blue circles) in the vicinity of the identified peak. This refinement significantly sharpens the detection signal. The peak of the refined scan shifts slightly and centers precisely at β=0.44. This value is in excellent agreement with the theoretical Onsager solution of $\beta_{c}\approx 0.4407$.

It is worth noting that the magnitude of the indicator differs between the two scans due to its dependence on the step size δt (analogous to the scaling of a finite difference derivative). However, the location of the peak remains robust. This two-stage experiment demonstrates that our method can effectively locate phase transitions in large-scale systems with high precision, balancing computational cost with detection accuracy.

### 3.3. Classic *N* = 100 SK Model

For a finite-size spin glass system with no applied external field, the corresponding Hamiltonian is denoted as [23]

(11) $$E\left(s\right)=-\sum_{i<j}J_{ij}S_{i}S_{j},$$

where the coupling constants $J_{ij}$ are independent and identically distributed Gaussian random variables, and the order parameter of the system is described by the Edwards–Anderson order parameter:

(12) $$q={\langle {\langle s_{i}\rangle }^{2}\rangle }_{d},$$

in which 〈〉 refers to a thermal average and ${\langle \rangle }_{d}$ refers to an average over the spatial disorder.

We consider the Sherrington–Kirkpatrick (SK) model, where the coupling constants $J_{ij}$ follow a Gaussian distribution with zero mean and a variance of 1/N. The theoretical critical temperature of the system is $T_{c}=1.0$. As the system is cooled from high temperatures to $T<T_{c}$, it undergoes a continuous phase transition from the high-temperature disordered paramagnetic phase to the low-temperature spin-glass phase with geometric spin frustration. The low-temperature spin-glass phase manifests as a frustrated, disordered state with well-defined statistical regularities. A distinct cusp in the heat capacity at $T_{c}$ confirms that this phase transition is a second-order transition.

**Figure 4:**
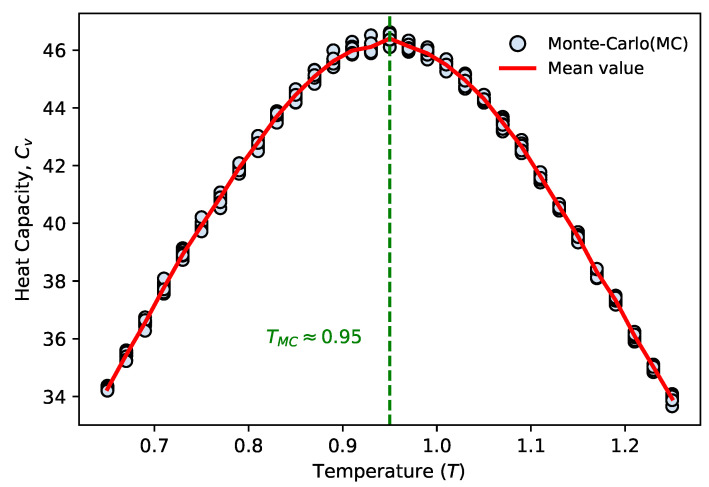
Inferring the phase transition of the SK model from limited sampling. Heat capacity obtained from annealed MCMC simulations, plotted as cyan data points. For each temperature point, 200,000 statistically independent samples are collected. Each MCMC chain covers 31 closely spaced temperature points across the range T=0.65 to T=1.25 with a temperature step of δT=0.02, and 10 consecutive independent replicate sampling runs are performed. The red solid line denotes the mean value of the 10 replicate runs at each temperature point. A pronounced peak of the specific heat is observed at $T_{MC}\approx 0.95$, corresponding to the finite-size critical temperature of the system.

We demonstrate our method using the SK model with a system size of N=100. We compute the heat capacity via annealed MCMC sampling to precisely determine the critical temperature of the system in the presence of finite-size effects. The results from 10 consecutive independent replicate sampling runs are presented in Figure 4, which shows that the heat capacity reaches a significant peak at $T_{MC}\approx 0.95$. We adopt this numerically determined critical temperature as the benchmark for evaluating our proposed method.

Owing to the randomness of the pairwise coupling constants $J_{ij}$ and competing frustration effects in spin-glass systems, the energy landscape is characterized by an exponentially large number of local minima (metastable states). This imposes fundamentally distinct requirements on the sensitivity of phase transition detection methods. The phase transition indicator given in (2) exhibits a critical lack of sensitivity for this class of phase transitions. It fails to effectively capture the phase transition signal and accurately locate the critical point, which clearly delineates the physical limits of the indicator’s applicability.

Targeting the intrinsic physical features of spin-glass phase transitions, we extend the detection framework and adopt a more universal indicator to validate our work:

(13) $$\sigma^{2}\left(lnp\right)=\langle \ln^{2}p\left(s\right)\rangle -{\langle \ln p\left(s\right)\rangle }^{2}.$$

For the probability p(s) obeying the Boltzmann distribution, the expression for this quantity is formally identical to the standard specific heat capacity formula:

(14) $$C_{v}=\beta^{2}\left(\langle E^{2}\rangle -{\langle E\rangle }^{2}\right).$$

We first perform a coarse scan over the temperature range of T∈[0.70,1.20] with a step size of δT=0.05 via annealed MCMC sampling (blue squares). Subsequently, we carry out a refined scan with a higher resolution δT=0.03 (green circles) to target and characterize the vicinity of the preliminarily identified peak. For each temperature point, 20,000 samples are used.

**Figure 5:**
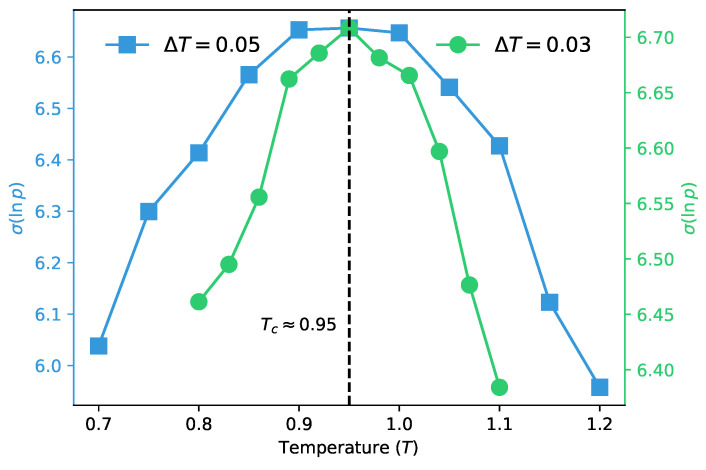
The detection results of the SK model. The dataset includes a coarse scan (δT=0.05) over a range of T∈[0.70,1.20] and a refined scan (δT=0.03) over a range of T∈[0.80,1.10] near the critical point. All configuration samples were generated using annealed MCMC. The dashed line indicates the critical point $T_{c}\approx 0.95$.

We perform density estimation on the samples using a densely connected model with autoregressive properties to handle one-dimensional data structures. We use a hidden layer autoregressive network with 100 neurons in the input and output layers and 500 neurons in the hidden layer, and then calculate the standard deviation of the log-probability of samples generated by this trained generative model. The results are presented in Figure 5, where (13) identifies a critical temperature in excellent quantitative agreement with the heat capacity peak position obtained from our MCMC sampling measurements. It successfully detects the paramagnetic-spin glass phase transition of the SK model, which demonstrates that even for the SK model with intricate non-trivial spin correlations, the autoregressive network can effectively learn the intrinsic statistical features of the target probability distribution from a limited number of samples.

## 4. Discussion

The core advantage of our approach lies in its agnosticism to the underlying physical mechanism. In many complex systems, such as spin glasses or topological phases of matter, the appropriate order parameter is often unknown or difficult to define analytically. Standard supervised learning methods require labeled data (e.g., ordered vs. disordered), which introduces human bias. In contrast, our method operates as a “generalized susceptibility” detector. By quantifying the rate of change of the probability distribution (via the derivative of the KL divergence), the indicator I(t) naturally captures the drastic reorganization of the configuration space at the phase boundary. Furthermore, the introduction of the absolute-value indicator ensures that the detection signal remains robust against sign cancellations and independent of the direction of parameter tuning. This places our method within the broader context of information-theoretic phase detection [39,40], yet with a distinct advantage: the explicit use of autoregressive generative models allows for exact likelihood computation, avoiding the estimation errors common in other variational bounds.

Looking forward, the versatility of this framework opens several promising avenues. Since the method relies strictly on sampled data, it is immediately applicable to experimental data (e.g., snapshots from cold atom experiments or electron microscopy) where the Hamiltonian is unknown. Extending this indicator to systems driven by multiple control parameters (e.g., temperature and external field simultaneously) or applying it to detect quantum phase transitions in wavefunction snapshots represents a straightforward yet impactful generalization of this work.

## 5. Conclusions

In this article, we have introduced a data-driven, unsupervised framework for detecting phase transitions by bridging deep generative modeling with statistical mechanics. Unlike traditional approaches that rely on pre-defined order parameters (e.g., magnetization) or supervised labels, our method identifies critical points solely by monitoring the intrinsic sensitivity of the probability distribution to control parameters. We validated this framework on the 2D ferromagnetic Ising model across distinct experimental setups: (i) an 8×8 triangular lattice with periodic boundaries using MCMC sampling, and (ii) a 16×16 square lattice with open boundaries using Tensor Network (PEPS) sampling. We further perform numerical experiments on the SK model via MCMC sampling. The paramagnetic-spin glass phase transition in this model involves intricate many-body correlations and cannot be characterized by any simple local order parameter. By calculating the standard deviation of the log-probability of samples, we obtain a critical point that is in excellent quantitative agreement with that determined from MCMC sampling measurements. A series of experiments spanning different lattice structures, boundary conditions and data sources conclusively demonstrate the robustness and generality of our proposed method.

Conventional ML methods, such as PCA and diffusion maps (DM), project high-degree-of-freedom systems onto a low-dimensional coordinate space via dimensionality reduction, which is implemented by calculating dynamical distances or custom-defined metric distances. They then identify dominant eigenvectors capturing strong correlations to uncover the intrinsic structure of the input data. Our proposed method follows a fundamentally distinct paradigm for information compression. Instead of explicitly searching for specific order parameters, our approach leverages the intrinsic autoregressive properties of density estimation and generative frameworks to enable the autoregressive network to autonomously learn complex spin-dependent correlations. Crucially, by leveraging autoregressive networks for density estimation, we successfully circumvent the intractable partition function, enabling direct access to the thermodynamic information encoded in raw configurations.

## Data Availability

A Python 3.10.9 implementation of our algorithm is available at https://github.com/XiyuZhou334/detect-phase-transition-by-GL (accessed on 27 November 2025).

## Appendix A. Several Indicators for Phase Transition Detection

Beyond the specific divergence-based indicator proposed in the main text, several other information-theoretic measures can potentially quantify the distinctness between phases. These measures provide a broader context for understanding phase transitions as geometric changes in the manifold of probability distributions [41].

### Appendix A.1. Distance-Based Measures

The Total Variation (TV) distance provides a straightforward metric for the distinguishability of two distributions *p* and *q*. In the context of phase transitions, it measures the non-overlap between configurations at different parameters:

(A1) $$I_{TV}=\frac{1}{2}\sum_{s}\left|p_{t}\left(s\right)-p_{t+\delta t}\left(s\right)\right|.$$

Another prominent measure is the Jensen–Shannon (JS) divergence, which is a symmetrized and smoothed version of the KL divergence. It is bounded and arguably more numerically stable in certain regimes:

(A2) $$I_{JS}=\frac{1}{2}KL\left(p_{t}\|M\right)+\frac{1}{2}KL\left(p_{t+\delta t}\|M\right),$$

where $M=\frac{1}{2}\left(p_{t}+p_{t+\delta t}\right)$ is the average distribution. Both TV and JS distance can serve as alternative candidates for detecting phase boundaries where the distribution undergoes abrupt changes.

### Appendix A.2. Theoretical Connection to Fisher Information

While the above measures operate on discrete parameter steps, the Fisher Information (FI) represents the differential limit of distributional sensitivity for continuous parameters. It provides a theoretical baseline for understanding generalized susceptibility [42,43].

Assuming the probability distribution p(s|t) is normalized and satisfies regularity conditions, we start with the identity

(A3) $$\int p\left(s|t\right)\frac{\partial \ln p(s|t)}{\partial t}ds=\frac{\partial }{\partial t}\int p\left(s|t\right)ds=\frac{\partial }{\partial t}\left(1\right)=0.$$

Taking the derivative of (A3) with respect to *t* yields

(A4) $$\int p\left(s|t\right){\left(\frac{\partial \ln p(s|t)}{\partial t}\right)}^{2}ds+\int p\left(s|t\right)\frac{\partial^{2}\ln p\left(s|t\right)}{\partial t^{2}}ds=0.$$

This leads to the well-known relationship between the variance of the score and the curvature of the log-likelihood:

(A5) $$E_{s∼p(\cdot |t)}\left[{\left(\frac{\partial \ln p(s|t)}{\partial t}\right)}^{2}\right]=-E_{s∼p(\cdot |t)}\left[\frac{\partial^{2}\ln p\left(s|t\right)}{\partial t^{2}}\right].$$

The Fisher Information F(t) is defined as this quantity:

(A6) $$F\left(t\right)=E_{s∼p(\cdot |t)}\left[-\frac{\partial^{2}\ln p\left(s|t\right)}{\partial t^{2}}\right].$$

In the context of statistical mechanics, the Fisher Information acts as a generalized susceptibility, measuring the local curvature of the statistical manifold [44,45]. While our proposed indicator in the main text utilizes the absolute magnitude of log-probability changes to capture critical fluctuations robustly, the Fisher Information offers a complementary continuous-limit perspective. In our experiments, calculating the discrete approximation of FI also yields peaks at phase boundaries, validating the information-geometric nature of the transition.

## Appendix B. Sampling the 2D Ising Model via 2D Tensor Networks

In our 16×16 square-lattice experiments, we bypass traditional Markov Chain Monte Carlo (MCMC) simulations and generate uncorrelated, high-fidelity samples directly using the Tensor Network State (TNS) formalism [37]. Unlike quantum systems that typically require contracting a double-layer network (representing the bra and ket), for classical statistical models such as the Ising model, the partition function *Z* corresponds to the full contraction of a single-layer Tensor Network.

### Appendix B.1. Mapping the Ising Model to Tensor Networks

The probability of a spin configuration s in the 2D Ising model follows the Boltzmann distribution:

(A7) $$\begin{array}{cc} P(s) & =\frac{1}{Z}e^{\beta J\sum_{\langle ij,kl\rangle }s_{ij}s_{kl}}=\frac{1}{Z}\prod_{\langle ij,kl\rangle }e^{\beta Js_{ij}s_{kl}}. \end{array}$$

We decompose the nearest-neighbor interaction term into a matrix product. Define the interaction matrix *M* as

(A8) $$\begin{array}{cc} \; & \;\;\;\;+1\;\;\;-1 \end{array}$$

(A9) $$\begin{array}{cc} M= & \left(\begin{array}{cc} e^{\beta J} & e^{-\beta J}\\ e^{-\beta J} & e^{\beta J} \end{array}\right)\begin{array}{cc} \; & +1\\ \; & -1 \end{array} \end{array}$$

Since *M* is symmetric and positive definite, it can be decomposed as $M=VV^{T}$. Specifically, for the Ising model, the matrix *V* is given by

(A10) $$\begin{array}{c} V=\left(\begin{array}{cc} \sqrt{\cosh(\beta J)} & \sqrt{\sinh(\beta J)}\\ \sqrt{\cosh(\beta J)} & -\sqrt{\sinh(\beta J)} \end{array}\right). \end{array}$$

Using this decomposition, we construct a local rank-5 tensor $A^{\left[i,j\right]}$ at each lattice site. The tensor elements are determined by the physical spin state s∈{+1,−1} and four virtual indices (l,r,d,u) corresponding to the left, right, down, and up neighbors:

(A11) $$\begin{array}{c} A_{s,l,r,d,u}^{\left[i,j\right]}=V_{s,l}V_{s,r}V_{s,d}V_{s,u}. \end{array}$$

This construction effectively places the interaction weights onto the bonds of the network. This representation is known as a Projected Entangled Pair State (PEPS).

### Appendix B.2. Partition Function as Network Contraction

A key property of the PEPS representation is that the partition function, $Z=\sum_{s}e^{-\beta E(s)}$, can be computed by contracting the physical indices. Specifically, summing over all possible physical spin states is equivalent to contracting the physical index of every local tensor $A^{\left[i,j\right]}$ with the all-ones vector $1={\left(1,1\right)}^{T}$:

(A12) $$Z=\mathrm{Contract}\left(\mathrm{PEPS},\underset{i,j}{⨂}\left(\begin{array}{c} 1\\ 1 \end{array}\right)\right).$$

This contraction reduces the entire grid to a scalar value *Z*, which encodes the total statistical weight of the system.

### Appendix B.3. Autoregressive Sampling

We perform direct sampling in an autoregressive manner, analogous to the generation process in autoregressive generative models. The conditional probability for the *k*-th spin $s_{k}$ given the previous configuration $s_{<k}$ is

(A13) $$\begin{array}{c} P\left(s_{k}|s_{<k}\right)=\frac{P(s_{1},\ldots ,s_{k})}{P(s_{1},\ldots ,s_{k-1})}=\frac{Z(s_{1},\ldots ,s_{k})}{Z(s_{1},\ldots ,s_{k-1})}. \end{array}$$

where $Z(s_{1},\ldots ,s_{k})$ represents a partial contraction of the network where the first *k* sites are fixed to specific spin values, while the remaining sites $s_{>k}$ are summed over (i.e., contracted with $1={\left(1,1\right)}^{T}$).

To efficiently compute these contractions row-by-row, we employ the Boundary Matrix Product State (BMPS) method. We utilize a variational boundary truncation scheme rather than the traditional SVD-based truncation. This approach significantly accelerates the contraction process for large batches of samples while maintaining high numerical precision.
